# Methanethiol Consumption and Hydrogen Sulfide Production by the Thermoacidophilic Methanotroph *Methylacidiphilum fumariolicum* SolV

**DOI:** 10.3389/fmicb.2022.857442

**Published:** 2022-03-28

**Authors:** Rob A. Schmitz, Sepehr S. Mohammadi, Timo van Erven, Tom Berben, Mike S. M. Jetten, Arjan Pol, Huub J. M. Op den Camp

**Affiliations:** ^1^Department of Microbiology, Radboud Institute for Biological and Environmental Research, Radboud University, Nijmegen, Netherlands; ^2^Environmental Chemistry, Institute of Biogeochemistry and Pollutant Dynamics, ETH Zürich, Zurich, Switzerland

**Keywords:** *Methylacidiphilum*, methanotrophs, methanethiol, hydrogen sulfide, thermoacidophile, sulfur cycle

## Abstract

Methanotrophs aerobically oxidize methane to carbon dioxide to make a living and are known to degrade various other short chain carbon compounds as well. Volatile organic sulfur compounds such as methanethiol (CH_3_SH) are important intermediates in the sulfur cycle. Although volatile organic sulfur compounds co-occur with methane in various environments, little is known about how these compounds affect methanotrophy. The enzyme methanethiol oxidase catalyzing the oxidation of methanethiol has been known for decades, but only recently the *mtoX* gene encoding this enzyme was identified in a methylotrophic bacterium. The presence of a homologous gene in verrucomicrobial methanotrophs prompted us to examine how methanotrophs cope with methanethiol. Here, we show that the verrucomicrobial methanotroph *Methylacidiphilum fumariolicum* SolV consumes methanethiol and produces H_2_S, which is concurrently oxidized. Consumption of methanethiol is required since methanethiol inhibits methane oxidation. Cells incubated with ∼15 μM methanethiol from the start clearly showed inhibition of growth. After depletion of methanethiol, growth resumed within 1 day. Genes encoding a putative methanethiol oxidase were found in a variety of methanotrophs. Therefore, we hypothesize that methanethiol degradation is a widespread detoxification mechanism in methanotrophs in a range of environments.

## Introduction

The potent greenhouse gas methane (CH_4_) is one of the key components of the global carbon cycle (Dean et al., 2018). Methane is emitted in large amounts from a variety of natural sources (e.g., wetlands, geothermal environments, and termites) and from industrial and agricultural sources (Etiope, 2009; Brune, 2010; Bridgham et al., 2013; Heede, 2014; Dean et al., 2018). Microorganisms living in natural or man-made environments can respire methane both aerobically (Hanson and Hanson, 1996; Murrell and Jetten, 2009) and anaerobically (Knittel and Boetius, 2009; Ettwig et al., 2010; Haroon et al., 2013). Aerobic methanotrophs are either members of the subphyla Alpha- and Gammaproteobacteria or the phylum Verrucomicrobia (Dunfield et al., 2007; Pol et al., 2007a; Islam et al., 2008; Op den Camp et al., 2009; van Teeseling et al., 2014; Schmitz et al., 2021). These verrucomicrobial methanotrophs are found in acidic geothermal habitats such as fumaroles and mudpots (Dunfield et al., 2007; Pol et al., 2007a; Islam et al., 2008; Erikstad et al., 2019; Awala et al., 2021). They share a low pH optimum (2.0–3.5) and all known isolates are part of the genus *Methylacidimicrobium* (optimum growth temperature at 30–50°C) or the genus *Methylacidiphilum* (optimum growth temperature at 50–60°C).

Methylotrophs are organisms that use reduced one-carbon compounds as energy and carbon source (Chistoserdova and Kalyuzhnaya, 2018). Methanotrophs are a special type of methylotrophs that possess a methane monooxygenase to oxidize methane to methanol (CH_3_OH) (Ross and Rosenzweig, 2017). Interestingly, verrucomicrobial methanotrophs possess the gene homolog *mtoX*, which was recently revealed to encode a copper-dependent methanethiol oxidase (MTO) in *Hyphomicrobium* sp. VS (Eyice et al., 2018). The presence of this gene suggests that the one-carbon compound methanethiol (CH_3_SH) could be a source of energy, carbon and sulfur (Eyice et al., 2018). Methanethiol is a foul-smelling volatile organic sulfur compound (VOSC) primarily degraded by microorganisms and a key intermediate of the global sulfur cycle (Lomans et al., 2002; Schäfer and Eyice, 2019). Moreover, methanethiol is toxic to animals and VOSCs in general are known to impact the environment in various ways, for instance through acid precipitation (Roman et al., 2016; Schäfer and Eyice, 2019; Kiragosyan et al., 2020; Maddry et al., 2020). Still, little is known about the effect of methanethiol on microorganisms (van den Bosch et al., 2009).

In nature, multiple biotic pathways lead to the production of methanethiol (Schäfer and Eyice, 2019). In marine systems, plankton produce the osmolyte dimethylsulfoniopropionate (DMSP), which can be degraded to methanethiol (Kiene, 1996). In both anoxic and oxic environments, the methylation of H_2_S and the degradation of sulfur-containing amino acids lead to methanethiol production (Lomans et al., 2001; Eyice et al., 2018). Moreover, methanethiol is produced from the degradation of dimethylsulfide (DMS) and dimethylsulfoxide (DMSO) and several methylotrophic methanogens were shown to grow on methanethiol and DMS (Finster et al., 1992; Lomans et al., 1999; Lyimo et al., 2000; Lyimo et al., 2009; Schäfer et al., 2010). The presence of methanethiol in acidic geothermal environments from which verrucomicrobial methanotrophs were isolated from is unresolved. These environments are characterized by emissions of various sulfur compounds such as H_2_S (Schmitz et al., 2021). Methanethiol can be formed abiotically (Heinen and Lauwers, 1995; Reeves et al., 2014) and in addition, biotic methanethiol production by Archaea in acidic environments has been observed (Baumler et al., 2007).

The gene encoding a putative MTO is found in all known verrucomicrobial methanotrophs (Schmitz et al., 2021). Several *Hyphomicrobium* strains were shown to degrade methanethiol using MTO to oxidize methanethiol to formaldehyde (CH_2_O), H_2_S and hydrogen peroxide (H_2_O_2_) (Suylen et al., 1987; Eyice et al., 2018). Interestingly, methylotrophs such as those of the genus *Hyphomicrobium* are effectively applied in biofilters to remove VOSCs from polluted industrial air (Pol et al., 1994; Pol et al., 2007b). On the contrary, very little is known about the mechanism through which methanotrophs cope with VOSCs such as methanethiol. Here, we show that methanethiol has an inhibitory effect on methanotrophy. *Methylacidiphilum fumariolicum* SolV grown on methane has a prolonged lag phase in the presence of methanethiol. *M. fumariolicum* SolV can degrade low concentrations of methanethiol, leading to the production and concurrent oxidation of H_2_S.

## Materials and Methods

### Chemostat Cultivation of *Methylacidiphilum fumariolicum* SolV on Methanol

*Methylacidiphilum fumariolicum* SolV isolated from a hot and acidic mud pool near Naples (Italy) was grown in a continuous bioreactor under methanol limitation. The medium composition and chemostat operation were performed as described before by Picone et al. (2020), without the addition of ethane. Briefly, the cells (OD_600_∼0.9 or 1.0) grew in a 300 mL chemostat at 55°C and pH 2.2 in medium supplemented with 50 mM methanol at a dilution rate D of 0.013 h^–1^. To grow the cells continuously, 3.9 mL medium per hour and 10.6 mL gas per minute (10% O_2_ (v/v) and 5% CO_2_ (v/v) in argon) were added.

### Preparation of Cell Fractions

Cell fractions of *M. fumariolicum* SolV were obtained as described before (Schmitz et al., 2020). Briefly, cells were lysed using a French pressure cell and the crude extract (CE) was obtained after centrifugation at 10,000 × *g* for 10 min at 4°C. Subsequently, the CE was centrifuged at 137,000 × *g* for 1 h at 4°C, leading to the separation of the soluble proteins in the supernatant (soluble fraction, SF) and the membrane proteins in the pellet (membrane fraction, MF). The pellet was subsequently homogenized, mixed with buffer containing the detergent n-dodecyl-β-D-maltoside and again centrifuged at 137,000 × *g* for 1 h at 4°C to obtain the solubilized membrane fraction (SMF) in the supernatant.

### Batch Incubations and Gas Chromatography

Batch incubations were performed in 120 mL serum bottles containing 10 mL cells (OD_600_∼0.9 or 1.0) and air. The bottles were closed with a rubber stopper and incubated at 55°C and 350 rpm with different concentrations of methanethiol, methane, or hydrogen sulfide. Methanethiol and methane were obtained from pure stocks, whereas hydrogen sulfide was prepared by mixing sodium sulfide with hydrochloric acid in a closed bottle to create hydrogen sulfide in the gas phase. To quantify methanethiol and hydrogen sulfide, 100 μl from the headspace of the bottles was injected with a glass Hamilton syringe into a gas chromatograph (7890B GC systems Agilent technologies, Santa Clara, CA, United States) equipped with a Carbopack BHT100 glass column (2 m, ID 2 mm) and a flame photometric detector (FPD) (Pol et al., 2018). Methane was measured as described before (Mohammadi et al., 2019). The areas obtained through GC injections were used to calculate the methanethiol, hydrogen sulfide and methane concentrations using standard curves. It was experimentally determined that the methanethiol concentration in the liquid is about 1.5 times higher than the methanethiol concentration in the gas phase at 55°C. Dry weight of the cells was determined as described by Picone et al. (2020).

### Growth Experiments on Methane and Methanethiol

To investigate growth of *M. fumariolicum* SolV on methane in the presence of methanethiol, cells from the methanol-limited continuous culture were diluted to an OD_600_ of 0.01 in a sterilized 120 mL serum bottle, containing 10 mL medium and a headspace containing air, CO_2_ (5%), CH_4_ (7.5%), and with or without 1200 nmol methanethiol. All experiments were performed in triplicates. The optical density of the culture and the consumption of methanethiol and methane were routinely measured.

### Phylogenetic Analysis

A representative set of genomes of alpha- and gammaproteobacterial methanotrophs was obtained from GenBank. The MTO sequence of *Hyphomicrobium* sp. VS (Eyice et al., 2018) was blasted against these proteobacterial genomes and *Candidatus* Methylomirabilis, using 10e-3 as the e-value threshold. Putative MTO sequences were added to the tree of Eyice et al. (2018), which already contained sequences of the verrucomicrobial methanotrophs *Methylacidiphilum fumariolicum* SolV and *Methylacidiphilum infernorum* V4. Sequences were aligned using Muscle 3.8.1551 (Madeira et al., 2019) and the tree was calculated using RAxML 8.2.10 (Stamatakis, 2014) with the rapid bootstrapping method and the PROTGAMMALGF substitution model. SignalP v. 5.0 was used to predict the cellular location of the putative MTO of 77 different methanotrophs (Almagro Armenteros et al., 2019).

## Results

### *Methylacidiphilum fumariolicum* SolV Consumes Low Concentrations of Methanethiol

**FIGURE 1:**
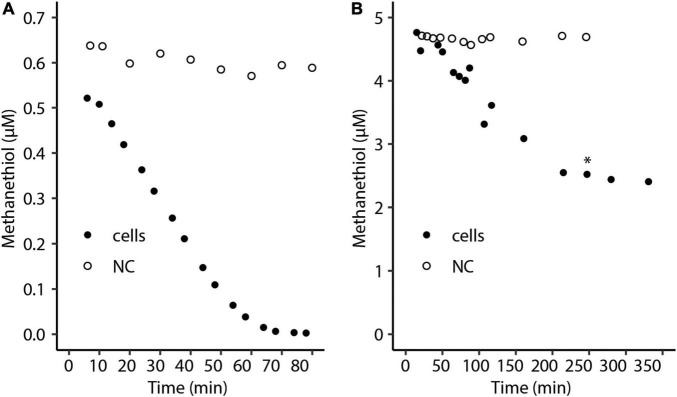
Consumption of a liquid concentration of approximately **(A)** 0.5 μM and **(B)** 5 μM methanethiol by *Methylacidiphilum fumariolicum* SolV cells over time. The asterisk indicates the time point at which additional oxygen was added. NC, negative control with medium only.

To show that strain SolV is able to oxidize methanethiol, cells from the methanol-limited continuous bioreactor (OD_600_∼0.9) were used for activity tests with different methanethiol concentrations in batch cultures. At a starting liquid concentration of approximately 0.5 μM methanethiol, the substrate was completely consumed (Figure 1A). Cells of *M. fumariolicum* SolV incubated with a starting liquid concentration of approximately 5 μM methanethiol consumed this compound at a constant rate of 0.58 nmol ⋅ min^–1^ ⋅mg DW^–1^ in the initial phase of the incubation (Figure 1B). However, after about half of the initial methanethiol amount was consumed, the consumption rate of methanethiol severely decreased, suggesting inhibition. Addition of oxygen did not enhance methanethiol consumption, excluding that oxygen was limiting (Figure 1B). When cells were incubated with an initial concentration of approximately 2.5 μM, the substrate was fully consumed. Therefore, the cells seem unable to completely consume methanethiol concentrations of approximately 3 μM or higher.

We also determined the affinity constant (K_*s*_) and V_*max*_ using non-inhibitory methanethiol concentrations in a range from 30 to 400 nmol (120-ml serum bottles, 10 ml cell suspension). The control, medium and methanethiol without cells, did not show oxidation of methanethiol. With SolV cells consumption rates of up to 1.7 nmol ⋅ min^–1^ ⋅ mg DW^–1^ were measured. From the data a highest rate of methanethiol consumption (V_*max*_) of about 2.3 nmol ⋅ min^–1^ ⋅ mg DW^–1^ and an affinity constant (K_*s*_) of about 0.1 μM was calculated.

We further tested the methanethiol oxidation by feeding it to a methanol limited continuous culture (D = 0.013 h^–1^). We observed an increase in OD_600_ from 1.04 to 1.14 (about 10% increase), and the highest consumption rate of methanethiol was measured at 0.21 nmol ⋅ min^–1^ ⋅ mg DW^–1^, which is about 1% of the methanol consumption rate. Moreover, similar to our observations in the batch activity experiments, we found that when methanethiol concentrations rose above about 3 nmol per ml gas in the head-space (<4.5 μM in the liquid) of the reactor, a part of the fed methanethiol was detected in the gas outlet of the reactor. Reactor performance became unstable pointing to inhibition.

### Methanethiol Adversely Affects Methanotrophy and Growth

**FIGURE 2:**
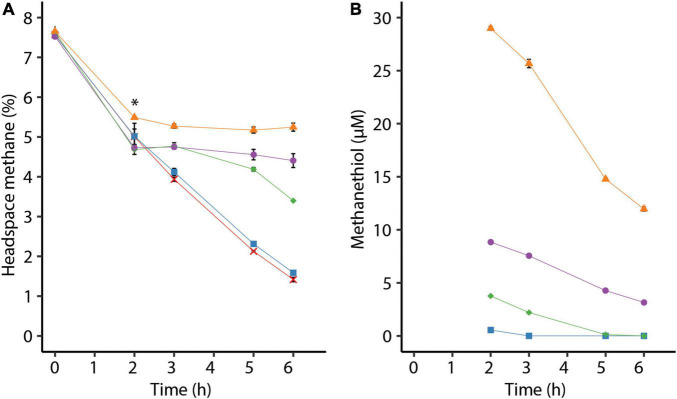
**(A)** Consumption of methane by *Methylacidiphilum fumariolicum* SolV cells over time and **(B)** decrease of methanethiol over time due to microbial and chemical degradation. The asterisk in panel **(A)** indicates the time point at which different amounts of methanethiol were added, leading to liquid methanethiol concentrations shown in panel **(B)**. To the positive control (red crosses) no methanethiol was added. Error bars indicate standard deviation (*n* = 3).

To investigate the effect of methanethiol on methanotrophy, cells from the methanol-limited continuous culture were transferred to serum bottles with 7.5% methane in the headspace. After 2 h, different amounts of methanethiol were added to create initial liquid concentrations of approximately 1–29 μM methanethiol. A liquid concentration of about 1 μM methanethiol does not affect methane oxidation and is depleted within an hour (Figures 2A,B). Methane oxidation of cells to which higher amounts of methanethiol were added were impeded at least temporarily (Figures 2A,B). When methanethiol was added to create a liquid concentration of approximately 4 μM, methane oxidation by *M. fumariolicum* SolV continued after more than half the amount of methanethiol was degraded (Figure 2). In the presence of approximately 9 μM methanethiol in the liquid, methane oxidation was inhibited but seems to resume when the concentration methanethiol had dropped below 4 μM. Interestingly, cells that were pre-incubated for 2 h with methane oxidized higher concentrations of methanethiol than cells that were not pre-incubated in batch (Figures 1B, 2B). When incubated with a liquid concentration of about 29 μM methanethiol, methane oxidation did not restore within 4 h (Figure 2A). Since methanethiol consumption continued after 4 h (Figure 2B), methane oxidation may still resume after methanethiol concentrations dropped below 5 μM.

**FIGURE 3:**
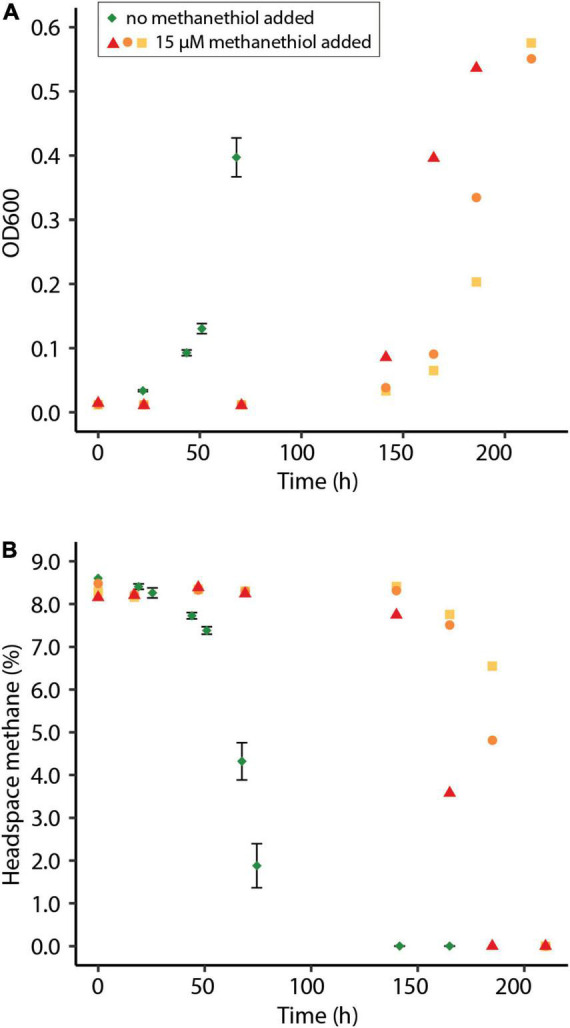
**(A)** Optical density measured at 600 nm (OD_600_) of methane-oxidizing *Methylacidiphilum fumariolicum* SolV cells in serum bottles and **(B)** the percentage methane in the headspace of the bottles over time. Green diamonds indicate average of positive controls to which no methanethiol was supplemented. Error bars indicate standard deviation (*n* = 3). Red triangles, orange dots, and yellow squares indicate incubations to which 1200 nmol methanethiol was added at the start of the experiment, to create approximately 15 μM methanethiol in the liquid. Methanethiol was depleted after 71 h. Experiments were performed in triplicate.

To observe the effect of methanethiol on growth of *M. fumariolicum* SolV on methane, cells from the continuous bioreactor were diluted in growth medium to OD_600_∼0.01 and grown in batch cultures. Cultures incubated with both methane and methanethiol showed a prolonged lag phase, compared to the control with methane only (Figure 3A). Cells incubated with 1200 nmol methanethiol (∼15 μM in the liquid) from the start clearly showed inhibition of growth with large variations between replicates (Figure 3). After depletion of methanethiol, growth resumed within one day. Cells incubated without methanethiol had a growth rate (μ) of 0.060 h^–1^ and a doubling time (T_*d*_) of 11.5 h, whereas cells incubated with 15 μM methanethiol (depleted after 71 h) had a μ of 0.043 ± 0.003 h^–1^ (T_*d*_ of 16.4 ± 1.0 h). It seems that regular growth starts after the high initial concentration of methanethiol is converted. The observed decrease in methanethiol is due a combination of microbial degradation and chemical degradation, as the methanethiol concentration in sterile medium decreases over time as well, leading to the production of mainly dimethyldisulfide (Supplementary Figure 1).

### *Methylacidiphilum fumariolicum* SolV Produces and Oxidizes Hydrogen Sulfide

**FIGURE 4:**
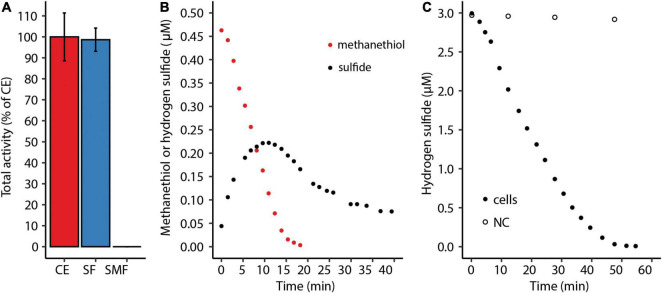
**(A)** Total methanethiol-degrading activity of the soluble fraction (SF) and the solubilized membrane fraction (SMF) as percentage of the total activity of the crude extract (CE). Error bars indicate standard deviations (*n* = 2). **(B)** Methanethiol consumption and H_2_S production by crude extract of *Methylacidiphilum fumariolicum* SolV. **(C)** Consumption of 3 μM H_2_S by *M. fumariolicum* SolV cells in a serum bottle over time. NC, negative control with medium only.

The putative methanethiol oxidase encoded by *M. fumariolicum* SolV is predicted to be a cytoplasmic protein by SignalP 5.0. Nevertheless, proteins could be predicted to be cytoplasmic based on amino acid sequence and still be associated with the membrane (Schmitz et al., 2020). To determine the cellular location of methanethiol consumption, the soluble proteins were separated from the membrane proteins using ultracentrifugation. Clearly, the entire capacity to degrade methanethiol is found in the soluble fraction, suggesting that the putative methanethiol oxidase could be responsible for the observed methanethiol degradation (Figure 4A). Interestingly, during methanethiol consumption by the crude extract the stoichiometry of methanethiol to H_2_S was never 1:1, caused by simultaneous production and consumption of H_2_S (Figure 4B). H_2_S oxidation was enhanced when methanethiol becomes depleted, suggesting substrate competition (Figure 4C). When H_2_S was added directly instead, a liquid concentration of 3 μM H_2_S is completely consumed.

### Genes Encoding Putative Methanethiol Oxidases Are Found in a Range of Methanotrophs

**FIGURE 5:**
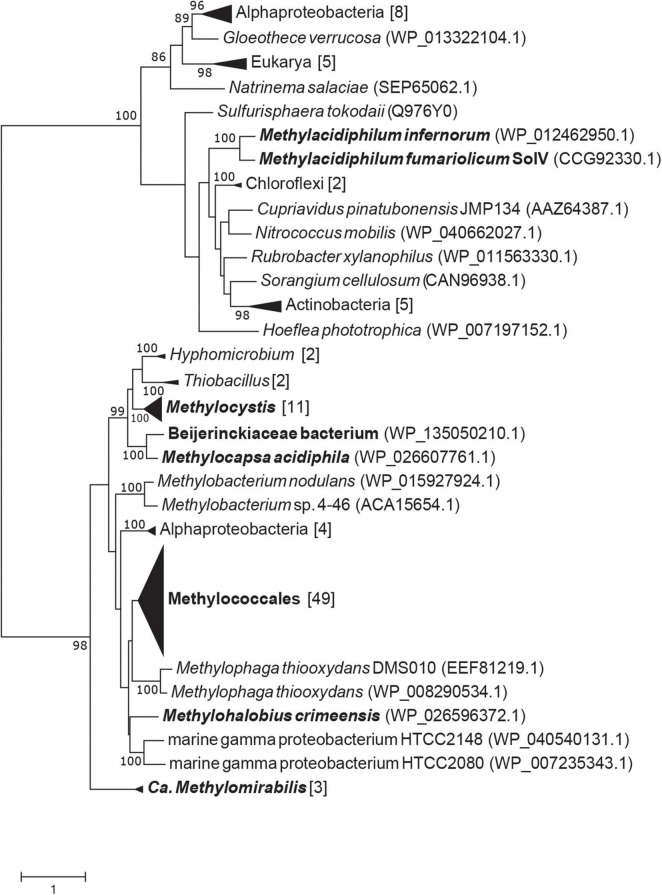
RAxML maximum likelihood tree of putative methanethiol oxidase amino acid sequences from methanotrophs. Bootstrap values above 80 are shown, based on 500 replicates. Black names indicate methanotrophic clades or strains.

After the discovery of the gene that encodes MTO in *Hyphomicrobium* sp. VS (annotated as selenium-binding protein 56) it became apparent that this gene is found in a wide range of environments (Eyice et al., 2018). Interestingly, specific mutations in the gene encoding MTO in humans cause extra-oral halitosis since methanethiol cannot be degraded (Pol et al., 2018). The putative MTO of *M. fumariolicum* SolV is 28% identical (43% positives; 1e-29) in amino acid sequence to MTO of *Hyphomicrobium* sp. VS (KY242492.1) and 36% identical (53% positives; 2e-89) to MTO of *Homo sapiens*. *mtoX* is present in all known verrucomicrobial methanotrophs, suggesting the capacity of methanethiol consumption as was shown for *M. fumariolicum* SolV in this study. Interestingly, we detected putative MTO in a range of methanotrophs. Strains that possess pMMO and/or sMMO in combination with a putative MTO were found in the alphaproteobacterial family *Beijerinckiaceae* and genera *Methylobacterium*, *Methylocapsa*, and *Methylocystis*, and in the gammaproteobacterial genera *Crenothrix*, *Methylobacter*, *Methylocaldum*, *Methylococcus*, *Methylicorpusculum*, *Methyloglobus*, *Methylohalobius*, *Methylomagnum*, *Methylomarinum*, *Methylomicrobium*, *Methylomonas*, *Methyloprofundus*, *Methylosarcina*, *Methylospira*, *Methyloterricola*, *Methylotetracoccus*, and *Methylotuvimicrobium* (Figure 5). In addition, putative MTO was found in the genus Candidatus *Methylomirabilis* sp., known for anaerobic methane oxidation through an intra-aerobic pathway (Ettwig et al., 2010; Versantvoort et al., 2018). Interestingly, all MTO homologs are predicted be periplasmic proteins, except for those found in verrucomicrobial methanotrophs and *Methylospira mobilis*, *Methylocaldum szegediense*, and *Methylotuvimicrobium alcaliphilum*.

## Discussion

In this study we have shown that the thermoacidophilic methanotroph *Methylacidiphilum fumariolicum* SolV consumes methanethiol, which leads to the production and subsequent consumption of H_2_S. All known verrucomicrobial methanotrophs encode for a putative cytoplasmic methanethiol oxidase, which could be responsible for the observed methanethiol consumption. Verrucomicrobial methanotrophs may utilize the methanethiol oxidase to detoxify methanethiol, since methanotrophy and growth on methane are inhibited by this organic sulfur compound. The methanethiol consumption rate is about 0.33% of methane consumption rates reported before (Mohammadi et al., 2017).

Methanethiol is an important volatile organic sulfur compound in the global sulfur cycle (Lomans et al., 2002). Recently, methanethiol was shown to be produced through thermogenic processes in seafloor hydrothermal systems (Reeves et al., 2014). Furthermore, it was shown that methanethiol can be formed abiotically from H_2_S, CO_2_, and H_2_ (Heinen and Lauwers, 1995). In view of this, the occurrence of methanethiol production in acidic geothermal environments in which verrucomicrobial methanotrophs thrive is very likely. The observation that *M. fumariolicum* SolV produces H_2_S from the degradation of methanethiol is in line with studies of MTO in *Hyphomicrobium* sp. EG and sp. VS and *Thiobacillus thioparus* (Suylen et al., 1987; Gould and Kanagawa, 1992; Eyice et al., 2018) and humans (Pol et al., 2018). Also in these organisms produced H_2_S is simultaneously oxidized. Alternatively, recently microorganisms were found to methylate methanethiol and produce dimethylsulfide aerobically and anaerobically (Lomans et al., 2001; Carrión et al., 2017). However, *mddA* encoding a methyltransferase catalyzing this reaction is absent in verrucomicrobial methanotrophs. Interestingly, *mtoX* homologs encoding methanethiol oxidase are found in proteobacterial methanotrophs of various genera. In addition, a study showed that the facultative methanotroph *Sphingopyxis* sp. MD2, isolated from a landfill in South Korea, is able to degrade methanethiol through an uninvestigated mechanism (Lee et al., 2012). However, molecular evidence of culture purity is lacking and the *pmoA* gene sequence is 98% identical to that of *Methylocystis* sp. 39 (AJ459045). In addition, several other methanotrophic strains were shown to be inhibited by methanethiol rather than be stimulated by it (Börjesson, 2001; Lee et al., 2011; Lee et al., 2015). Considering the toxicity of methanethiol, possessing an MTO could be beneficial in environments where methane and methanethiol are known to co-occur, such as aquatic sediments and landfills (Lomans et al., 1997; Kim et al., 2005; Mayr et al., 2020).

Methanethiol clearly inhibits methane oxidation and growth of *M. fumariolicum* SolV, but the underlying mechanism is unclear. In addition, cells of strain SolV are unable to completely consume methanethiol at concentrations above 3 μM, pointing to inhibition by toxic degradation product as described before (Suylen et al., 1987). Sulfide is a toxic compound that is known to inhibit the respiratory chain and several enzymes by binding to the active site (Bagarinao, 1992; Landry et al., 2021). In microorganisms, methanethiol could have a similar mode of inhibition. Alternatively, if MTO is the enzyme dedicated to the degradation of methanethiol in verrucomicrobial methanotrophs, one or more products of this catalysis, formaldehyde (CH_2_O), sulfide, and hydrogen peroxide (H_2_O_2_), could account for the observed inhibition. The product formaldehyde is a central intermediate in carbon assimilation in the majority of methylotrophs (Suylen et al., 1987; Chistoserdova, 2011). Through the serine cycle and the RuMP cycle in Alpha- and Gammaproteobacteria, formaldehyde is fixed whereas verrucomicrobial methanotrophs oxidize formaldehyde to CO_2_ through an unresolved pathway. MTO was shown to oxidize H_2_S, but verrucomicrobial methanotrophs also possess a sulfide:quinone oxidoreductase (SQR) that could be dedicated to this catalysis (Suylen et al., 1987; Schmitz et al., 2021). In addition, exogenous H_2_S and H_2_S produced by MTO in the cytoplasm could be used for sulfur assimilation. Finally, catalases detoxify hydrogen peroxide to water and oxygen, but these enzymes are not found in all verrucomicrobial methanotrophs, although enzymes with similar functions could be used, such as peroxidases (Schäfer et al., 2010; Schmitz et al., 2021).

The finding that cell pre-incubated with methane for 2 h can oxidize much higher concentrations of methanethiol is interesting. When methanethiol is supplemented to these energized cells, methane oxidation ceases while methanethiol is degraded. This observation may indicate that also pMMO is involved in the degradation of methanethiol. Partitioning of pMMO in methanethiol degradation cannot be observed through cell fractionation, as this procedure disrupts pMMO activity. pMMO is a monooxygenase and therefore needs reducing equivalents to catalyze a reaction. Accordingly, the cells pre-incubated with methane have synthesized a relatively high concentration of reducing equivalents that could subsequently be used to reduce pMMO for the degradation of methanethiol. Indeed, Börjesson (2001) showed that methane oxidation in landfill soils is inhibited by methanethiol and that methane and methanethiol seem to compete for the same enzymes, which could be pMMO. Whether pMMO is indeed involved in methanethiol degradation in methanotrophs remains to be investigated and could be resolved by using a pMMO-specific inhibitor.

In conclusion, we show that *M. fumariolicum* SolV is able to consume methanethiol and concurrently produce and consume hydrogen sulfide. H_2_S is known to be emitted from terrestrial volcanic ecosystems such as mud pools and oxidation of this compound can lead to severe acidification of the environment (Spiro et al., 1992; Lomans et al., 2002). Emissions of methanethiol from the habitat of verrucomicrobial methanotrophs are unknown, but it may be produced both chemically and by microorganisms. Methanethiol has an inhibitory effect on methane oxidation, which is presumably alleviated through MTO in *M. fumariolicum* SolV. Since putative MTOs are found in a range of methanotrophs, we propose these enzymes to be a widespread mechanism for methanethiol degradation in methanotrophs. Future studies are needed to observe whether methanethiol has an inhibitory effect on methanotrophs in general and whether cells can be adapted to conserve energy from methanethiol.

## Data Availability Statement

The raw data supporting the conclusions of this article will be made available by the authors, without undue reservation.

## Author Contributions

RS, SM, TE, AP, and HO designed the project and experiments. RS, SM, TE, and AP performed the experimental work. RS, SM, and AP maintained the chemostat cultures. TB performed the phylogenetic analysis. RS, SM, AP, and HO performed data analysis and data interpretation. RS, SM, and HO wrote the manuscript with feedback from TE, TB, MJ, and AP. HO, AP, and MJ supervised the research. All authors contributed to the article and approved the submitted version.

## Conflict of Interest

The authors declare that the research was conducted in the absence of any commercial or financial relationships that could be construed as a potential conflict of interest.

## Publisher’s Note

All claims expressed in this article are solely those of the authors and do not necessarily represent those of their affiliated organizations, or those of the publisher, the editors and the reviewers. Any product that may be evaluated in this article, or claim that may be made by its manufacturer, is not guaranteed or endorsed by the publisher.
