# Supplementary material for: Methanethiol Consumption and Hydrogen Sulfide Production by the Thermoacidophilic Methanotroph Methylacidiphilum fumariolicum SolV
Source: Front Microbiol. 2022 Mar 28;13:857442. doi: 10.3389/fmicb.2022.857442 (PMC9003020; doi:10.3389/fmicb.2022.857442)
Supplement: Supplementary file 1 [file Data_Sheet_1.PDF]

Supplementary Material

Methanethiol consumption and hydrogen sulfide production by the thermoacidophilic methanotroph *Methylacidiphilum fumariolicum* SolV

Rob A. Schmitz[1,2], Sepehr S. Mohammadi[1], Timo van Erven[1], Tom Berben[1], Mike S.M. Jetten[1], Arjan Pol[1], Huub J.M. Op den Camp[1*]

[1]Department of Microbiology, Radboud Institute for Biological and Environmental Research, Radboud University, Nijmegen, the Netherlands

[2]Environmental Chemistry, Institute of Biogeochemistry and Pollutant Dynamics, ETH Zürich, Switzerland

*** Correspondence:** h.opdencamp@science.ru.nl

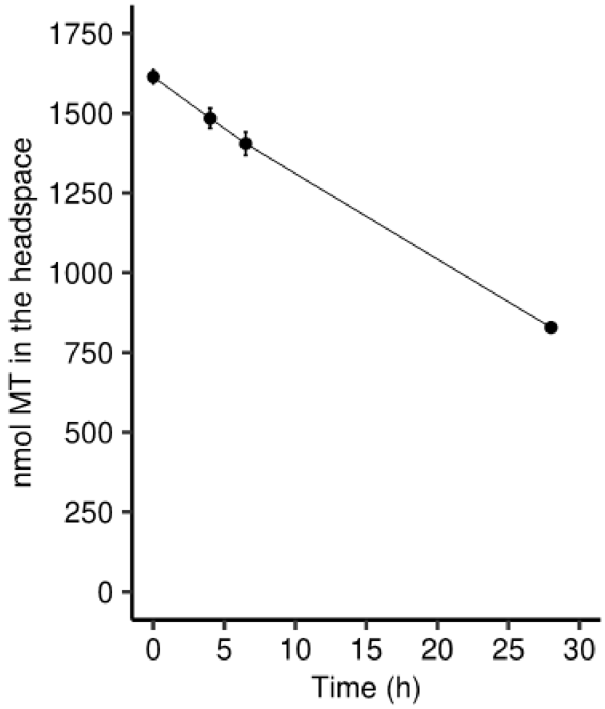

Supplementary Figure S1: Decrease of methanethiol over time in a 120 mL serum bottle containing 10 mL sterile growth medium. Error bars indicate standard deviation ($n = 3$).
